# Thioaptamer Conjugated Liposomes for Tumor Vasculature Targeting

**DOI:** 10.18632/oncotarget.261

**Published:** 2010-05-07

**Authors:** Aman P. Mann, Rohan C. Bhavane, Anoma Somasunderam, Brenda Liz Montalvo-Ortiz, Ketan B. Ghaghada, David Volk, René Nieves-Alicea, K. Stephen Suh, Mauro Ferrari, Ananth Annapragada, David G. Gorenstein, Takemi Tanaka

**Affiliations:** ^1^ Department of Nanomedicine, University of Texas Health Science Center at Houston, 1825 Hermann Pressler, Houston, Texas, 77030; ^2^ School of Biomedical Informatics, University of Texas Health Science Center at Houston, 7000 Fannin, Houston, Texas, 77030; ^3^ Institute of Molecular Medicine, University of Texas Health Science Center at Houston, 1825 Hermann Pressler, Houston, Texas, 77030; ^4^ Department of Pharmaceutical Sciences, Thomas Jefferson University,130 South 9th Street, Philadelphia, PA, 19107; ^5^ The John Theurer Cancer Center, Hackensack University Medical Center, Hackensack, New Jersey, 07601; ^6^ The Methodist Hospital Research Institute, Houston, Texas, 77030

**Keywords:** cancer, therapy, liposomes, nanoparticles, tumor vasculature

## Abstract

Recent developments in multi-functional nanoparticles offer a great potential for targeted delivery of therapeutic compounds and imaging contrast agents to specific cell types, in turn, enhancing therapeutic effect and minimizing side effects. Despite the promise, site specific delivery carriers have not been translated into clinical reality. In this study, we have developed long circulating liposomes with the outer surface decorated with thioated oligonucleotide aptamer (thioaptamer) against E-selectin (ESTA) and evaluated the targeting efficacy and PK parameters. *In vitro* targeting studies using Human Umbilical Cord Vein Endothelial Cell (HUVEC) demonstrated efficient and rapid uptake of the ESTA conjugated liposomes (ESTA-lip). *In vivo*, the intravenous administration of ESTA-lip resulted in their accumulation at the tumor vasculature of breast tumor xenografts without shortening the circulation half-life. The study presented here represents an exemplary use of thioaptamer for targeting and opens the door to testing various combinations of thioaptamer and nanocarriers that can be constructed to target multiple cancer types and tumor components for delivery of both therapeutics and imaging agents.

## INTRODUCTION

Treatment for solid tumors has largely relied on initial surgical intervention and subsequent scheduled cytotoxic chemotherapies. However, the vast majority of malignancies have proven to be resistant to these approaches, partially due to the requisite dose limitations for preventing adverse effects on normal tissues. Recent advances in multi-functional drug delivery systems provide insight into development of nanoscale carriers for cancer treatment and imaging [1-3]. Current nanoscale delivery systems that are approved by FDA rely on passive targeting through leaky vessels, which is a hallmark of tumor vasculature [4, 5]. This class of nanoparticles improves the solubility, toxicity profile, and unfavorable pharmacokinetics of the chemotherapeutics. However, the therapeutic efficacy remains largely unchanged [6, 7]. Therefore, development of a tool to allow for constant and selective delivery of therapeutics is desirable. Active targeting can be achieved by efficient recognition of tumor specific antigens that are differentially expressed cell surface proteins between normal and tumor. An affinity-based interaction between the antigens and targeted ligand increase delivery efficacy by enhancing the retention of nanoparticles and elevating cellular uptake while minimizing side effects associated with potential off-targeting [1].

Among tumor components, tumor vasculature is an attractive target for the delivery of anti-cancer and imaging contrast agents. The concept of tumor vasculature targeting is substantially distinct from targeting cancer cells [1, 8]. When nanoparticles are administered intravenously to target cancer cells, they are required to physically cross over the layers of endothelial lining against oncotic pressure that is generated from inside the tumor microenvironment. In contrast, for vasculature targeted nanoparticles, the intended molecular targets on the endothelial cell surface are readily accessible to the circulating nanoparticles. Tumor vasculature is in an inflamed state and overexpresses several important factors such as adhesion molecules [9, 10]. E-selectin (CD62E, ELAM-1 or LECAM-2) is selectively expressed in inflamed vasculature in advanced tumors by cytokines that are secreted in the inflammatory tumor microenvironment [11-13]. The inducible nature of E-selectin in the inflammatory environment would allow for site specific delivery of nanoparticle to the inflamed tumor vasculature and possibly to tumor parenchyma via subsequent extravasation. We recently identified a thiophosphate-backbone modified oligonucleotide aptamer (“thio”aptamer) that binds E-selectin expressed on endothelial cells with high affinity and specificity [14]. In this study, we developed E-selectin thioaptamer conjugated stealth liposomes and analyzed physico-chemical properties, targeting efficiency, and pharmacokinetics. We herein report on 1) application of thioated aptamer for drug delivery, 2) tumor vasculature targeting, and 3) the effect of aptamer conjugation on pharmacokinetics of stealth liposomes.

## RESULTS

### Development of ESTA conjugated liposomes

**Figure 1:**
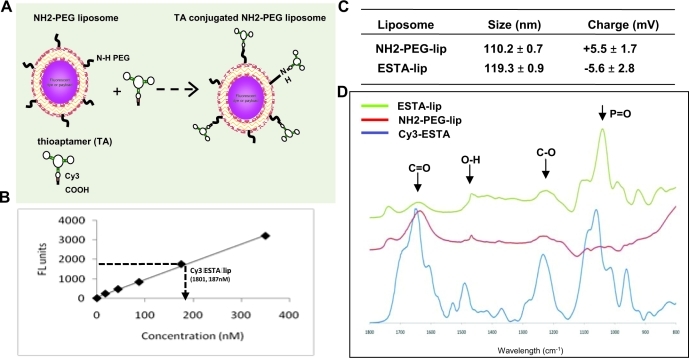
Physico-chemical properties of ESTA conjugated liposomes (A) Scheme of TA conjugation to liposome. (B) Quantification of ESTA conjugation on the liposomes. Fluorescence intensity of Cy3-labeled ESTA was plotted against ESTA concentration. Using the standard curve the number of ESTA molecules on ESTA-lip was estimated based on fluorescence measurement. (C) Size and charge of the liposomes. The size of the liposomes was measured using a dynamic light scattering (DLS) and the surface charge was measured by zeta potential. (D) FTIR spectrum of the non labeled-liposomes and ESTA-lips.

We first developed ESTA conjugated liposome (ESTA-lip) for effective vasculature targeting. The efficiency of conjugation was evaluated by coupling Cy3 labeled carboxylated ESTA (COOH-Cy3-ESTA) on amino PEGylated stealth liposome (NH2-PEG-lip) (A) and the intensity of red fluorescence was measured at anexcitation/ emission of 544/594 nm using a fluorimeter. A linear standard curve was generated from Cy3-labeled ESTA. Approximately 50% of the surface amino groups presenton the liposome were conjugated to the ESTA (~485 ESTA molecules conjugated to one liposome) (Fig. 1B). The physico-chemical properties of the liposomes wereanalyzed by a zeta-sizer and Fourier Transform InfraRed spectroscopy (FTIR). The size of NH2-PEG-lip was 110.2nm in average, and ESTA conjugation caused a slight increase of size of the liposomes (119.3 nm) (Fig. 1C). The analysis of zeta-potential showed that ESTA conjugation resulted in a substantial change of the surface charge of the NH2-PEG lip from +5.5 ± 1.7 mV to −5.6 ± 2.8 mV (Fig. 1C). This indicated that the positive charge of the amino groups on the liposome surface was substituted by the negatively charged ESTA. Additionally, the spectra acquired from FTIR analysis of the Cy3-ESTA-lip were compared to the NH2 PEG lip and ESTA (Fig. 1D). A reduction in peaks corresponding to C=O stretch (1650 cm^−1^) and C-O stretch (1230 cm^−1^) as compared to the non-reactive peak corresponding to P=O bending (1040 cm-1) was observed in ESTA-lip. The O-H bend in the COOH-ESTA (1480 cm^−1^) appeared slightly shifted and partially reactive due to the contribution of O-H from water. Taken together, these data demonstrated the successful conjugation of COOH-Cy3-ESTA to the NH2-PEG-lip.

### Binding of ESTA-lip to inflamed endothelial cells

**Figure 2:**
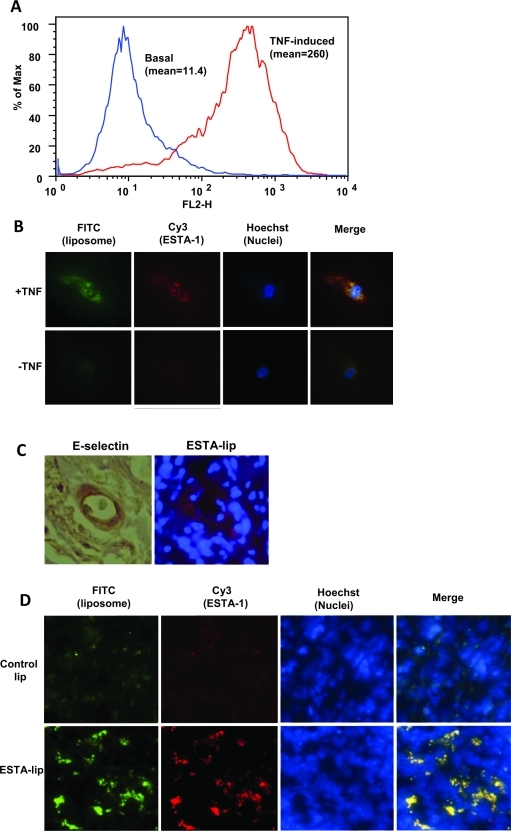
E-selectin dependent binding of ESTA-lip on E-selectin expressing cells (A) Induction of E-selectin expression on the HUVEC cells. HUVEC were stimulated with TNF-α (10 ng/ml) for 4 hours. Cells were dissociated with 5mM EDTA and then incubated with FITC labeled E-selectin antibody. Surface fluorescence was measured by flow cytometry and averaged for three separate experiments. (B) ESTA-lip binding to the HUVEC expressing E-selectin. HUVEC was incubated with TNF-α (10 ng/ml) for 3 hours to induce E-selectin expression. The cells were further incubated with Cy3-ESTA-lip-FITC (10 nM) for 2 hours. All images were captured at the same exposure condition for comparison. The final images shown are representative images (at the final magnification: x600) from five random fields of at least three independent experiments. Blue, Hoechst 33342; Red, ESTA; Green, FITC-lip. (C) Immunohistochemical analysis for E-selectin expression on the vasculature of mouse bearing breast xenograft tumor derived from MDA-MB-231. ESTA-lip-Rhodamine were intravenously injected and the tumor was dissected 5 hr after the injection and frozen sections were generated to identify the localization of the liposomes by using fluorescent microscope. (D) ESTA-lip accumulation in tumor parenchyma after 48 hr after the injection. Red, lip-Rhodamine; Blue, Hoechst 33342.

To test binding efficiency of ESTA-lip *in vitro*, HUVEC were stimulated with TNF-α to induce E-selectin expression on the cell membrane. TNF-α treatment increased the expression of E-selectin more than 20-fold when compared to untreated cells (Fig. 2A). The TNF-α treated cells were used to test the ability of the Cy3-ESTA-lip-FITC (Cy3-ESTA conjugated liposome containing FITC) to bind to E-selectin on the HUVEC cell surface. The cells treated with Cy3-ESTA-lip-FITC at a concentration of 10 nM showed intense FITC and Cy3 fluorescence in the cells stimulated with TNF-α when compared with un-stimulated controls (Fig. 2B). The merged fluorescence of FITC and Cy3 suggest that the Cy3-ESTA-lip-FITC were internalized after binding to E-selectin on the cell surface. This co-migration of both thioaptamer and liposome was observed within 24 hours (data not shown). Regardless of TNF stimuli, un-conjugated liposomes as negative control only showed minor interaction to the cells and the fluorescent signal was almost undetectable after normalization (supplemental figure 1). To further examine selective binding of ESTA-lip to E-selectin *in vivo*, ESTA-lip-Rhodamine was i.v. injected into mice bearing breast tumor xenografts (Figure 2C). The expression level of E-selectin was upregulated on the tumor vasculature of xenograft tumors, predominantly expressed in or neighboring regions of tumor stroma (Fig. 2C, left). The mouse bearing tumors received an injection of ESTA-lip-Rhodamine via tail vain, and the mice were sacrificed 5 h post-injection to harvest the tumor and the major organs. Intravenous bolus injection of the ESTA-lip-Rhodamine (3 mg of liposome in 100 μl saline) resulted in an accumulation of red fluorescence on the tumor vasculature (Fig. 2C, right), while minimal level of fluorescence was detected around the vessel area when un-conjugated-lip was injected (data not shown). In contrast, ESTA conjugation did not affect accumulation of liposomes in the sinusoidal organs (data not shown). These data demonstrated that ESTA-lip preferentially binds to E-selectin expressing human and mouse endothelial cells *in vitro* and *in vivo*. Furthermore, 48 h after the injection, ESTA-lip accumulation in the tumor parenchyma was increased markedly (Fig. 2D), suggesting that enhanced tumor vasculature targeting may facilitate subsequent extravasation of the liposomes into tumor parenchyma. Interestingly, the appearance of speckled patterns obtained from these experiments was similar to that of ESTA alone [14]. This speckled pattern might be due to either intracellular vesicle localization or simply a reflection of clustered and discontinuous E-selectin expression pattern on the cell surface [15]. Several studies suggest that E-selectin internalizes and undergoes a recycling following membrane sorting. In addition, our 3-D confocal imaging analysis of E-selectin expressing endothelial cells demonstrated intracellular localization of ESTA, suggesting internalization of ESTA (unpublished). Although binding kinetics were not measured, 50% of the coverage of the liposomes containing 1.1% amine-PEG with ESTA was sufficient to produce high-retention kinetics (Fig. 2B and C). In this study, we formulated the liposomes to contain 1.1% of amino-PEG, although the ratio can be modified up to 5% to further enhance the amounts of ESTA bioconjugation to increase binding specificity and affinity.

### Pharmacokinetics of ESTA-lip

Pharmacokinetics of the liposomes was then analyzed by taking whole blood at different time points after a single intravenous bolus administration of the ESTA-lip-Rhodamine or NH2-PEG-lip into 10 weeks old FBV mice. The fluorescence intensity originated from Rhodamine in the plasma of the treated mice over the untreated mice was assessed. Following a one compartmental model, the pharmacokinetics parameters were determined. There was no significant difference in the initial volume of distribution between two types of liposomes (4.2 ± 0.2 mL for NH2-PEG-lip *vs*. 4.2 ± 0.4 mL for ESTA-lip). On the other hand, ESTA conjugation resulted in a decrease in the clearance rate (CL) (0.12 ± 0.03 μg.h for NH2-PEGlip vs. 0.09 ± 0.01 μg.h for ESTA-lip), and in turn led to an increase of the area under the curve (AUC) (24848 ± 4897 μg.h/mL for NH2-PEG-lip *vs*. 33349± 4236 μg.h/mL for ESTA-lip) and slight extension of the circulation half-life (T_1/2_) (23 ± 4 h for the NH2-PEG-lip *vs*. 32 ± 7 h for ESTA-lip). This data supported that the presence of thioaptamer ligands (ESTA) on the surface of stealth liposomes does not cause a reduction of the bioavailability.

**Table:** 

	Kel (hr^−1^)^a^	T_1/2_ (hr)^b^	Vd (mL)^c^	AUC (mg.hr/mL)^e^	CL (mL/hr)^d^
PEG-lip	0.030 ± 0.004	24 ± 4	4.2 ± 0.2	24848 ± 4897	0.12 ± 0.03
ESTA-lip	0.022 ± 0.004	32 ± 7	4.2 ± 0.4	33349 ± 4236	0.09 ± 0.01
^a^ The elimination rate constant (kel) was obtained from the linear regression plot of the log Cp vs time curves. ^b^The half life (T_1/2_) was calculated using the formula: T(1/2)= 0.693/kel. ^c^Volume of distribution (Vd) was calculated using the formula: Vd= dose/C_o_, where Co is the extrapolated initial concentration. ^d^Area under the curve was calculated from the formula: AUC= C_o_/Kel, ^e^The Clearance (CL) was calculated using the formula: CL= Kel × Vd					

## DISCUSSION

Many targeted delivery systems have been developed using different types of nanoparticles and ligands for both imaging and drug delivery [16-20]. While antibodies have been the mainstay for active targeting, the use of antibodies or peptide based ligands for nanocarriers remains a challenge [21-23]. For example, it is difficult to control the orientation of antibody on the surface of nanocarriers by chemical conjugation since there are a number of reactive groups within a molecule, and such random conjugation may lead to unexpected off-target effects as well as poor targeting performance. More importantly, antibody conjugation to carriers causes a reduction in serum half-life due to the clearance by phagocytes in tissues and peripheral circulation [21-23]. The use of humanized or fragmented antibodies may address this issue; however, such strategies appear to be neither cost effective nor practical. For this reason, we selected thioaptamer. Aptamers are structurally distinct RNA and DNA oligonucleotides that have been shown to mimic protein-binding molecules, and yet exhibit high (nM to pM) binding affinity and selectivity [24], and can be an attractive alternative over antibody or peptide ligands based on their biological and chemical properties including, 1) small size, 2) high affinity binding, 3) low toxicity and immunogenicity, 4) simple and inexpensive chemical synthesis and modification process, and 5) ability to define the orientation during conjugation[25]. Among the aptamers that have been identified so far, two DNA classes of aptamers against prostate-specific membrane antigen (PSMA) [26] and nucleolin [27] have been conjugated to nanoparticles, for the cancer cell specific targeted drug delivery, and demonstrated effective delivery of chemotherapeutic agent specifically to cancer cells [28, 29].

As expected, ESTA conjugation to stealth liposome did not compromise the T_1/2_, and rather caused a slight extension of the T_1/2_. The mechanisms underlying extended T_1/2_ of ESTA-lip is not clear. One possible explanation might be that the negative surface charge of ESTA-lip (-7.5 ± 0.9 mV) attributed from substitution of the surface amino groups might have reduced the interaction of ESTA-lip to the negatively charged immune cell surfaces. Nevertheless, our finding points to the direction that thioaptamer conjugation is not likely to cause a loss of stealth effect of nanoparticles. Active targeting of stealth liposome may allow for a reduction in the dose of administration and/or decrease toxicity for the chemotherapeutics. In this study, we focused on E-selectin targeted thioaptamer (ESTA) and stealth liposomes, however, the combinations of type of aptamer and nanoparticles are virtually unlimited for the development of desirable nanoparticles.

## MATERIAL AND METHODS

### Synthesis of Cy3-labeled carboxylated ESTA

ESTA (5'-CGCTCGGA*TCGA*TA*A*GCTTCGA*TCCCA*CTCTCCCGTTCA*CTTCTCCTCA*C GTCA*CGGA*TCCTCTA*GA*GCA*CTG-3', * indicates thioation) was chemically synthesized in a DNA synthesizer (Expedite 8909, Applied Biosystems) using the standard phosphoramidite chemistry as described previously [30]. The 5' end of the ESTA was coupled with Cy3 phosphoramidite with the MMT protective group remaining and followed by the addition of 5'-carboxy modifier-C10 containing N-hydroxy succinimide ester protective group. After functionalization, ESTA was cleaved from the bead support and the protecting groups were removed with 0.4M methanolic NaOH at room temperature for 24-36 hr. The resultant ESTA was further purified and the concentration of the ESTA was determined by measuring the absorbance at 260 nm with a UV spectrophotometer.

### Preparation of ESTA conjugated liposome (ESTA-lip)

Amino-PEG liposomes were made with a lipid mixture (50 mM) consisting of DPPC, cholesterol, and DSPE-PEG (2000) Amine, in a 58.9: 40: 1.1 molar ratio, dissolved in ethanol and then hydrated with either; (1) 10 mM phosphate buffered saline to prepare empty liposomes or (2) 1 mM FITC solution for *in vitro* studies. This was then sequentially extruded using a Lipex thermoline extruder to prepare liposomes of approximately 100 nm. The FITC-liposomes were diafiltered through 500 kDa molecular weight cut-off membranes to remove un-encapsulated dye. Rhodamine tagged liposomes used for *in vivo* studies were extruded as above. DPPC: Chol: DSPE-PEG (2000) amine: Rhodamine-DHPE at 58.8: 40: 1.1 : 0.1 molar ratio were used. Carboxylated Cy3-labeled or unlabeled ESTA was conjugated to amino PEG liposomes by using carbodiimide chemistry with EDC and sulfo-NHS. Free ESTA was removed by dialysis.

### Characterization of ESTA-lips

The size and zeta potential of the ESTA conjugated liposome were measured using a ZetaPals instrument (Brookhaven Instruments). 2μl of liposomes were added to 1.4 mL of 10mM phosphate buffer (pH 7.3) and the analysis was conducted at room temperature (23°C) in triplicates. To quantify the concentration of the ESTA on the liposomes, Cy3 fluorescence from ESTA-lip was measured and compared with ESTA standard curve. Fourier transform infrared spectroscopy (FTIR) was preformed to assess the attachment of ESTA on the liposomes. Samples were diluted in de-ionized water and FTIR was preformed on a Nicolet 6700 (Thermo Scientific, Waltham, MA). A 2μL drop from each sample was placed on the diamond crystal and subjected to vacuum. Using a smart diamond crystal attenuated total reflection (ATR) accessory each sample was run for 150 scans at a resolution of 4 wave numbers.

### Induction of E-selectin expression in HUVEC cells

To induce E-selectin expression, the cells were stimulated with TNF-α (10 ng/ml) for 2 to 4 h. To determine E-selectin expression on the endothelial cell surface, the cells were dissociated with 5 mM EDTA and then incubated with FITC labeled E-selectin antibody (Pharmingen). As a negative control, the same amount of normal IgG was used. The surface fluorescence was measured by a FACS Caliber. The experiments were repeated at least three times and data represent an average value.

### ESTA-lip binding to E-selectin expressing endothelial cells

100 nM of Cy3-ESTA conjugated liposomes encapsulating FITC (Cy3-ESTA-lip-FITC) were incubated with HUVEC that were treated or untreated with TNF-α for 2 hours at 37°C. As a negative control, NH2 PEG liposomes encapsulating FITC (NH2-PEG-lip) were used. Following 3 hours incubation, the cells were briefly washed with tissue culture media to remove the unbound liposomes and then incubated overnight. The cells were fixed with 4% paraformaldehyde, and the nuclei were counterstained with 1.0 μg/ml Hoechst 33342. The fluorescent signals were detected using TE2000-E, Nikon fluorescent microscope (x600 magnification) to determine the binding to the cells. All images were acquired under the same exposure conditions for the comparison of liposome binding to the cells.

### *In vivo* carcinoma mouse model

5-week old female athymic *nu/nu* nude mice (Charles River) were maintained in a VAF-barrier facility and all animal procedures were performed in accordance with the regulations in the Institutional Animal Care and Use Committee at the University of Texas Health Science Center at Houston. An orthotopic breast tumor was established as previously reported with minor modification. When tumors became palpable (approx. 200-300 mm^3^), either encapsulated ESTA-lip-rhodamine or NH2-PEG-lip (3 mg of total lipid in 100 μl of saline) was intravenously injected into mice via tail vein (n=3 per group). One day after the injection, the tumors were harvested and immediately mounted in OCT media for subsequent histological analysis. To examine tumor localization of liposomes, the frozen tissues were sectioned (8 μm thickness) and analyzed by fluorescent microscope. The frozen sections were also immunostained as previously reported [14].

### Pharmacokinetics of liposomes

Following a single intravenous bolus administration of the ESTA-Lip-Rhodamine or NH2-PEG-Lip (3 mg of total lipid in 100 μl of saline) into 10 weeks old FBV mice (n=3-4), whole blood was collected at different time points by cardiac puncture. The fluorescence intensity in the plasma (50 μl) was measured using a fluorimeter at 544/594 nm (excitation/emission wavelengths) to determine the pharmacokinetics parameters of each liposome. Plasma samples were also collected from untreated mice as a baseline.

## 


